# A high preoperative Glasgow prognostic score predicts a high likelihood of conversion from laparoscopic to open surgery in patients with colon cancer

**DOI:** 10.1007/s00464-018-6369-8

**Published:** 2018-07-25

**Authors:** Yoshimi Iwasaki, Mitsuru Ishizuka, Kazutoshi Takagi, Hiroyuki Hachiya, Norisuke Shibuya, Yusuke Nishi, Taku Aoki, Keiichi Kubota

**Affiliations:** Department of Gastroenterological Surgery, Dokkyo Medical University, 880 Kitakobayashi, Mibu, Tochigi 321-0293 Japan

**Keywords:** Colon cancer, Conversion, Glasgow prognostic score, Laparoscopic surgery, Risk factor

## Abstract

**Background:**

Although the use of laparoscopic resection for colon cancer (LRC) has been increasing, conversion to open surgery sometimes becomes necessary because of intraoperative difficulties. Although the Glasgow prognostic score (GPS) is well known to be a predictor of outcome in patients with various cancers, it is unclear whether the preoperative GPS can predict the need for conversion from laparoscopic to open surgery.

**Objective:**

To investigate factors predictive of conversion from laparoscopic to open surgery in patients with colon cancer.

**Methods:**

Data from 308 consecutive patients who underwent LRC between January 2006 and March 2017 were retrospectively enrolled. Preoperative clinical factors in patients who had undergone LRC were compared between conversion and non-conversion groups, and multivariate regression analysis was performed to identify preoperative factors that might predict conversion from laparoscopic to open surgery.

**Results:**

Among 308 patients who had undergone LRC, conversion to open surgery was necessary in 28 (9.1%). Sixteen of the latter patients (6.8%) had GPS 0 (among a total of 234) and 6 (11.5%) had GPS 1 (among a total of 52). The proportion of patients with GPS 2 who required conversion was 27.2% (6/22), which was significantly higher than for those with GPS 0 or 1. Multivariate analysis demonstrated that GPS 2 (odds ratio [OR] 3.352; 95% confidence interval [CI] 1.049–10.71; *p* = 0.041) and preoperative ileus (OR 7.405; 95% CI 2.386–22.98; *p* = 0.001) were independent factors predictive of conversion from laparoscopic to open surgery.

**Conclusions:**

A high preoperative GPS is an independent factor predictive of conversion from laparoscopic to open surgery in patients with colon cancer.

The use of laparoscopic surgery has become widespread because of its minimal invasiveness. Randomized controlled trials have revealed that the initial and long-term outcomes of laparoscopic resection for colon cancer (LRC) are not inferior to those of conventional open surgery [1–4]. On the basis of this evidence, LRC has been widely adopted, even for patients with advanced colon cancer. However, recent studies have demonstrated that patients who undergo conversion from laparoscopic to open surgery have poorer initial and long-term outcomes than those who undergo conventional surgery [3, 5, 15]. The proportion of patients undergoing elective LRC requiring conversion from laparoscopic to open surgery is reported to be 10–23% [6, 7]. In order to reduce this conversion rate, adequate patient selection is required.

The Glasgow prognostic score (GPS) is an inflammation-based prognostic system involving only serum C-reactive protein (CRP) and albumin, and widely considered to be one of the most useful scoring systems for prognostication of patients with various cancers [8–10]. However, no previous studies have investigated the relationship between the GPS and conversion from laparoscopic to open surgery.

Here, we hypothesized that patients with a high GPS might have not only a poorer prognosis but also a higher likelihood of requiring conversion from laparoscopic to open surgery than those with a low GPS. To verify this hypothesis, we conducted a retrospective cohort study to investigate whether the preoperative GPS in patients with colon cancer is predictive of conversion from laparoscopic to open surgery.

## Patients and methods

For this study, we enrolled 308 patients who had undergone LRC at Dokkyo Medical University Hospital between January 2006 and March 2017. None of them had undergone laparoscopic surgery for rectal cancer or benign diseases. Patients who had undergone LRC combined with hepatectomy for hepatic metastasis were excluded. The 308 patients with colon cancer were reviewed retrospectively, and preoperative and intraoperative data for the same period were collected.

We considered that LRC was indicated for patients with colon cancer that had been diagnosed clinically as T1–T4a/N0-2/M0-1 on the basis of preoperative colonoscopy and abdominal computed tomography (CT). If preoperative ileus had been sufficiently improved by decompression, LRC was approved for such patients by preoperative conference. Even when LRC had been approved, the final decision was confirmed after the patients had furnished informed consent.

For univariate analysis using logistic regression, patients were divided into two age groups (≥ 65/< 65) on the basis of the definition of “elderly” by the World Health Organization [11]. Body mass index (BMI) was divided into two categories (≥ 25/< 25) on the basis of the definition of obesity [12]. Preoperative ileus was diagnosed when abdominal CT demonstrated colon or small bowel distension accompanied by symptoms such as nausea, vomiting, and abdominal pain.

Laboratory parameters including hemoglobin (Hgb) (upper physiological value, 12 g/dL), CRP (upper physiological value, 0.14 mg/dL), albumin (upper physiological value, 4.1 g/dL), and tumor markers such as carcinoembryonic antigen (CEA) (upper physiological value, 5.0 ng/mL) and carbohydrate antigen 19-9 (CA19-9) (upper physiological value, 37 U/mL) were measured on the initial day to exclude signs of clinical improvement resulting from treatments such as blood transfusion or total parenteral nutrition (TPN).

The GPS was estimated as described previously [8, 9]. Patients with both an elevated CRP level (> 1.0 mg/dL) and hypoalbuminemia (< 3.5 g/dL) were allocated a score of 2. Patients with only one of these biochemical abnormalities were allocated a score of 1, and patients with neither were allocated a score of 0. Therefore, the cut-off values of CRP and albumin were determined on the basis of this definition.

Tumor location was determined by preoperative colonoscopy or barium enema and reconfirmed using intraoperative observation. The cecum, ascending colon, and transverse colon were defined as the right-sided colon, whereas the descending colon, sigmoid colon, and rectosigmoid colon were defined as the left-sided colon.

Conversion to open surgery was defined as follows: (1) An incision longer than 8 cm was required. Very large tumors that required an incision exceeding 8 cm were excluded on the basis of this criterion. (2) Open techniques were used. The decision to convert from laparoscopic to open surgery was finally made at the discretion of the surgeons. Therefore, tumor size was also divided into two categories (> 7/≤ 7 cm) for univariate analysis.

Data were expressed as the median with range. Differences between the two groups divided by the need for conversion (±) or the GPS (0–1/2) were analyzed using the Mann–Whitney *U* test or Chi-Squared test. Univariate and multivariate analyses were performed using logistic regression. Statistical analyses were performed using the SPSS statistical software package (version 23.0, IBM Co., New York, NY, USA) at a significance level of *P* < 0.05.

## Results

This study included 172 males and 136 females with a median age of 68 years (range 31–100 years); 234 had GPS 0, 52 had GPS 1, and 22 had GPS 2. Seventeen patients had preoperative ileus. Among them, 7 patients underwent preoperative decompression using long tube insertion (*n* = 5) and colonic stenting (*n* = 2). The other 10 received TPN instead of oral intake. Conversion from laparoscopic to open surgery was needed for 28 patients (9.1%). The conversion rate in patients with GPS 2 was 27.3%, and that for patients with GPS 0 and 1 was 6.8 and 11.5%, respectively. The conversion rate for patients with GPS 2 was significantly higher than that for patients with GPS 0 or 1. There were no significant differences between the two groups (conversion +/conversion −) in terms of age, gender, BMI, American Society of Anesthesiologists (ASA), presence of cardiopulmonary disease, previous laparotomy, preoperative Hgb and CEA levels, tumor location and size, and pathological tumor-node-metastasis (pTNM) stage and pathology. Preoperative ileus and very large tumors were observed more frequently in patients who required conversion than in those who did not (Table 1). The conversion rate in patients with preoperative ileus was 41.2% (7/17) and that in patients with very large tumors (> 7 cm) and with stages III and IV was 20.7% (6/29) and 14.4% (17/118), respectively.

**Table 1:** Characteristics of patients who underwent laparoscopic resection for colon cancer with or without conversion

Variables	Conversion (+) (*n* = 28)	Conversion (−) (*n* = 280)	*P*
Age (years)	69 (41–89)	67 (31–100)	0.537
Gender			
Male	17 (60.7)	155 (55.4)	
Female	11 (39.3)	125 (44.6)	0.586
BMI (kg/m^2^)	22.8 (16.3–32.6)	22.2 (13.3–37.6)	0.766
ASA grade			
1	6 (21.4)	55 (19.6)	
2	20 (71.5)	205 (73.2)	
3	2 (7.1)	20 (7.2)	0.974
Cardiopulmonary disease			
Absence	23 (82.1)	228 (81.4)	
Presence	5 (17.9)	52 (18.6)	0.926
Previous laparotomy			
Absence	18 (64.2)	196 (69.8)	
Presence	10 (35.7)	84 (30.2)	0.531
Preoperative ileus			
Absence	21 (75.0)	270 (96.4)	
Presence	7 (25.0)	10 (3.6)	< 0.001
CEA (ng/l)	3.7 (1–1450)	2.9 (1–3330)	0.439
CA19-9 (U/mL)	6 (2-396)	8 (2–12000)	0.648
Hgb (g/dL)	12.6 (7.6–16.9)	12.7 (4.9–17.1)	0.726
GPS			
0	16 (57.2)	218 (77.9)	
1	6 (21.4)	46 (16.4)	
2	6 (21.4)	16 (5.7)	0.005
Tumor location			
Right-sided colon	12 (42.9)	126 (45.0)	
Left-sided colon	16 (57.1)	154 (55.0)	0.828
Tumor size (mm)	38 (2–80)	37 (5–120)	0.418
Very large tumor (> 7 cm)			
Absence	22 (78.6)	257 (91.8)	
Presence	6 (21.4)	23 (8.2)	0.022
pTNM stage			
0	2 (7.1)	18 (6.4)	
I	5 (17.9)	83 (29.6)	
II	4 (14.3)	78 (27.9)	
III	13 (46.4)	80 (28.6)	
IV	4 (14.3)	21 (7.5)	0.128
Pathology			
Well	13 (46.4)	103 (36.8)	
Moderately	12 (42.9)	164 (58.5)	
Poorly	2 (7.1)	5 (1.8)	
Mucinous	1 (3.6)	8 (2.9)	0.173

Categorical variables were expressed as number (percent) and compared using Chi-Square test. Continuous variables were expressed as median (range) and compared using Mann–Whitney test

*ASA* American Society of Anesthesiologists, *BMI* body mass index, *CA19-9* carbohydrate antigen 19-9, *CEA* carcinoembryonic antigen, *GPS* Glasgow prognostic score, *pTNM* pathologic tumor, nodes, metastases

Patients with GPS 2 had a significantly lower BMI than those with GPS 0 or 1. The preoperative serum level of CEA was significantly higher in patients with GPS 2 than in those with GPS 0 or 1. However, there was no significant difference between the two groups (GPS 0 or 1/GPS 2) in the preoperative serum level of CA19-9. The preoperative Hgb level was significantly lower in patients with GPS 2 than in those with GPS 0 or 1. Although there were no significant differences between the two groups in tumor location and pathology, tumor size and pTNM stage were more advanced in patients with GPS 2 than in those with GPS 0 or 1. The median size of the tumor in patients with GPS 0 or 1, and those with GPS 2, was 3.5 and 5.1 cm, respectively (Table 2).

**Table 2:** Relationships between clinical characteristics and GPS

Variables	GPS 0 or 1 (*n* = 286)	GPS2 (*n* = 22)	*P*
Age (years)	67 (31–100)	74 (48–87)	0.065
Gender			
Male	161 (56.3)	11 (50.0)	
Female	125 (43.7)	11 (50.0)	0.567
BMI (kg/m^2^)	22.4 (13.3–37.6)	20.6 (15.9–30.7)	0.011
ASA grade			
1	59 (20.6)	2 (9.1)	
2	209 (73.1)	16 (72.7)	
3	18 (6.3)	4 (18.2)	0.067
Cardiopulmonary disease			
Absence	236 (82.5)	15 (68.2)	
Presence	50 (17.5)	7 (31.8)	0.095
Previous laparotomy			
Absence	199 (69.6)	15 (68.2)	
Presence	87 (30.4)	7 (31.8)	0.891
Preoperative ileus			
Absence	271 (94.8)	20 (90.9)	
Presence	15 (5.2)	2 (9.1)	0.447
CEA (ng/l)	2.9 (1.0–294)	6.2 (1.3–3330)	0.007
CA19-9 (U/mL)	7 (2–12000)	9 (2–1410)	0.234
Hgb (g/dL)	12.9 (4.9–17.1)	11.3 (7.4–13.8)	< 0.001
Tumor location			
Right-sided colon	126 (44.1)	12 (54.5)	
Left-sided colon	160 (55.9)	10 (45.5)	0.34
Tumor size (mm)	35 (2–120)	51 (22–105)	0.001
Very large tumor (> 7 cm)			
Absence	265 (92.7)	14 (63.6)	< 0.001
Presence	21 (7.3)	8 (36.4)	
pTNM stage			
0	19 (6.6)	1 (4.5)	< 0.001
I	87 (30.4)	1 (4.5)	
II	74 (25.9)	8 (36.4)	
III	88 (30.8)	5 (22.8)	
IV	18 (6.3)	7 (31.8)	
Pathology			
Well	109 (38.1)	7 (31.9)	0.805
Moderately	163 (57.0)	13 (59.1)	
Poorly	6 (2.1)	1 (4.5)	
Mucinous	8 (2.8)	1 (4.5)	

Categorical variables were expressed as number (percent) and compared using Chi-Square test. Continuous variables were expressed as median (range) and compared using Mann–Whitney test

*ASA* American Society of Anesthesiologists, *BMI* body mass index, *CA19-9* carbohydrate antigen 19-9, *CEA* carcinoembryonic antigen, *GPS* Glasgow prognostic score, *pTNM* pathologic tumor, nodes, metastases

Univariate analyses revealed that preoperative ileus (presence/absence), GPS (2/0,1), very large tumor (> 7/≤ 7 cm), and pTNM stage (III, IV/0, I, II) were associated with conversion from laparoscopic to open surgery (Table 3). Multivariate analysis using these four selected factors demonstrated that the presence of preoperative ileus and GPS 2 were independent factors predictive of conversion from laparoscopic to open surgery (Table 3).

**Table 3:** Univariate and multivariate analyses in relation to conversion to open surgery

Variables	Univariate analyses			Multivariate analysis		
	*P* value	Odds ratio	95% CI	*P* value	Odds ratio	95% CI
Age (≥ 65/< 65)	0.467	1.350	0.601–3.030			
Gender (female/male)	0.587	0.802	0.363–1.775			
BMI (> 25/≤ 25)	0.254	1.632	0.703–3.784			
ASA (III/I, II)	1.000	1.000	0.221–4.520			
Cardiopulmonary disease (presence/absence)	0.926	0.953	0.346–2.625			
Previous laparotomy (presence/absence)	0.532	1.296	0.574–2.926			
Preoperative ileus (presence/absence)	< **0.001**	9.000	3.109–26.06	**0.001**	7.405	2.386–22.98
CEA (> 5.0/≤ 5.0 ng/mL)	0.480	1.341	0.594–3.030			
CA19-9 (> 37/≤ 37 U/mL)	0.855	1.124	0.318–3.970			
Hgb (< 10/≥ 10 g/dL)	0.689	1.232	0.444–3.420			
GPS (2/0, 1)	**0.004**	4.500	1.600–12.66	**0.041**	3.352	1.049–10.71
Very large tumor (> 7/≤ 7 cm)	**0.029**	3.047	1.123–8.271	0.193	2.144	0.680–6.766
Tumor location (left/right)	0.828	1.091	0.498–2.391			
pTNM stage (III, IV/0, I, II)	**0.013**	2.739	1.235–6.075	0.132	1.929	0.821–4.536
Pathology (poorly, mucinous/well, moderately)	0.181	2.465	0.658–9.232			

Bold values indicate significance at *P* < 0.05

*ASA* American Society of Anesthesiologists, *BMI* body mass index, *CA19-9* carbohydrate antigen 19-9, *CI* confidence interval, *CEA* carcinoembryonic antigen, *GPS* Glasgow prognostic score, *pTNM* pathologic tumor, nodes, metastases

The reasons for conversion to open surgery are listed in Table 4. Major reasons for conversion were severe intra-abdominal adhesion (*n* = 9) and a narrow operative space (*n* = 9). Direct invasion to adjacent organs was an additional reason (*n* = 7), and pathological examination revealed that such direct invasion was diagnosed in only three patients. Other reasons for conversion were uncontrollable intraoperative bleeding (*n* = 2) and hypotension (*n* = 1). The conversion rate in patients with preoperative ileus was 41.2%. Two patients had both GPS 2 and preoperative ileus, and both underwent conversion to open surgery (data not shown).

**Table 4:** Reasons for conversion to open surgery

Reason	Number (%)
Adhesion	9 (32)
Narrow operative space	9 (32)
Direct invasion to adjacent organ	7 (25)
Bleeding	2 (7)
Intra-operative hypotension	1 (4)
Total	28 (100)

## Discussion

The GPS is a well-known inflammation-based prognostic system used in patients with various cancers. Forrest et al. first reported the prognostic use of the GPS in patients with non-small-cell lung cancer in 2004 [8, 9]. In 2007, McMillan et al. and Ishizuka et al. subsequently reported that a high preoperative GPS was able to predict poor outcome after resection of colorectal cancer [13, 14]. However, the relationship between the GPS and conversion from laparoscopic to open surgery has never been investigated. To our knowledge, this is the first retrospective study to have investigated the relationship between the preoperative GPS and conversion from laparoscopic to open surgery in patients with colon cancer. Our findings revealed that a high preoperative GPS was associated with conversion from laparoscopic to open surgery.

Although preoperative ileus, preoperative GPS 2, tumor size (> 7 cm), and stage (III, IV) were factors predictive of conversion from laparoscopic to open surgery, BMI, male gender, ASA, and previous laparotomy were not related to conversion from laparoscopic to open surgery in this study.

A meta-analysis of laparoscopic colorectal cancer surgery has concluded that laparoscopic resection was less likely to be completed in males and in patients with a high BMI [15]. It is well known that male gender, presence of rectal cancer, and a high BMI are factors predictive of conversion from laparoscopic to open surgery due to the narrow pelvic space and technical difficulties [16]. These factors were not considered in the present study because no rectal cancer patients were included and most patients had a lower BMI than those reported in western countries [17].

The same meta-analysis reported that laparoscopic resection was less likely to be completed in patients with locally advanced tumors and rectal tumors [15]. As a matter of course, locally advanced tumors such as those at stage III and IV, and also very large tumors, would have had a higher likelihood of necessitating conversion from laparoscopic to open surgery in the present series. However, tumors at stages III and IV, and those that were very large, were not significantly associated with such conversion. Multivariate regression analysis demonstrated that preoperative ileus and GPS 2 were independent risk factors for conversion to open surgery, rather than stage III and IV and very large tumors. This study is the only one so far to have considered not only gender, age, BMI, ASA, previous laparotomy, and cardiopulmonary disease, but also preoperative ileus and GPS; most previous studies that investigated conversion to open surgery in LRC did not consider these preoperative factors, including GPS [1, 2, 17, 18].

Although patients with preoperative GPS 2 had a lower BMI than those with GPS 0 or 1, the former showed a higher rate of conversion than those with GPS 0 or 1. A high GPS was defined as the presence of hypoalbuminemia and an increased serum level of CRP. It is well known that the serum levels of albumin and CRP are related to inflammation due to the influence of inflammatory cytokines such as IL-6 [19]. In addition, cancer progression is closely associated with inflammation induced by abundant pro-inflammatory cytokines [20]. Therefore, a high GPS may reflect not only cancer progression but also the systemic inflammatory response, rather than conventional tumor-related factors such as stage and a very large tumor size.

It has been reported that preoperative hypoalbuminemia was the most important factor predictive of conversion to open surgery in patients undergoing laparoscopic cholecystectomy for cholecystolithiasis [21]. A high GPS, which includes hypoalbuminemia, would be a very feasible indicator to predict conversion in laparoscopic surgery patients with malignant disease, because hypoalbuminemia would also be strongly associated with conversion in benign disease.

Preoperative ileus was a factor strongly predictive of conversion to open surgery in the present study. In fact, approximately 15–22% of patients with colorectal cancer have symptoms of acute obstruction [22, 23]. Most patients with bowel obstruction undergo open surgery or emergency surgery instead of laparoscopic surgery, because bowel obstruction is one of the most common reasons for conversion to open surgery [24].

Recent studies have revealed that colonic stenting, as a bridge to elective surgery, can contribute to not only an increased rate of laparoscopic surgery but also to a decreased conversion rate [25, 26]. Although most of the present patients with preoperative ileus were managed by TPN instead of oral intake, intraoperative bowel distension was not fully improved. Therefore, laparoscopic surgery in such patients would be more difficult than for those without preoperative ileus, and the rate of conversion to open surgery might be increased in such patients. Surprisingly, in the present study, the conversion rate was 100% in patients who had both preoperative ileus and GPS 2.

In conclusion, this retrospective study has demonstrated that the GPS is applicable for routine preoperative clinical assessment of patients with colon cancer and is useful for decision-making. Because a high preoperative GPS is associated with a high rate of conversion to open surgery, the GPS would be a feasible and valuable tool for surgical decision-making in colon cancer patients undergoing LRC.

## Abbreviations

ASA: American Society of Anesthesiologists
BMI: Body mass index
CA19-9: Carbohydrate antigen 19-9
CEA: Carcinoembryonic antigen
CRP: C-reactive protein
CT: Computed tomography
GPS: Glasgow prognostic score
Hgb: Hemoglobin
LRC: Laparoscopic resection for colon cancer
TNM: Tumor-node-metastasis
TPN: Total parenteral nutrition
